# 2,6-Bis[(*S*)-4-benzyl-4,5-dihydro-1,3-oxazol-2-yl]pyridine

**DOI:** 10.1107/S1600536811014061

**Published:** 2011-04-22

**Authors:** Konstanze Möller, Kathrin Junge, Anke Spannenberg, Matthias Beller

**Affiliations:** a Leibniz-Institut für Katalyse e. V. an der Universität Rostock, Albert-Einstein-Strasse 29a, 18059 Rostock, Germany

## Abstract

The commercially available title compound, C_25_H_23_N_3_O_2_, has been known since 1993 [Nesper *et al.* (1993 ▶). *Helv. Chim. Acta*, **76**, 2239–2249], but has not been structurally characterized until now. In the free ligand, the N atoms of both oxazoline rings point in opposite directions. The phenyl rings make dihedral angles of 30.56 (5) and 84.57 (3)° with the pyridine ring and 72.85 (3)° with each other.

## Related literature

For the synthesis, see: Nesper *et al.* (1993 ▶; 1996 ▶); Schaus & Jacobsen (2000 ▶); Towers *et al.* (2003 ▶); Meng *et al.* (2005 ▶); Hui *et al.* (2006 ▶). For crystal structures showing the same ligand coordinated to Pd(BF_4_)_2_ or AgBF_4_, see: Nesper *et al.* (1996 ▶); Provent *et al.* (1997 ▶). For applications in asymmetric catalysis, see: Desimoni *et al.* (2003 ▶); Tse *et al.* (2006 ▶). 
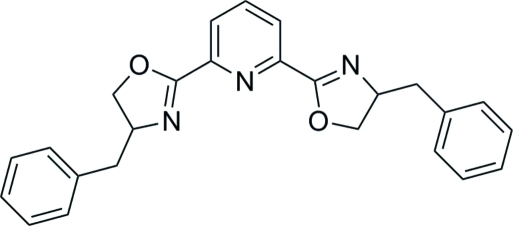


## Experimental

### 

#### Crystal data

C_25_H_23_N_3_O_2_
*M* *_r_* = 397.46
Orthorhombic, 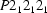

*a* = 7.0184 (2) Å
*b* = 13.2654 (3) Å
*c* = 21.5542 (8) Å
*V* = 2006.74 (10) Å^3^
*Z* = 4
Mo *K*α radiation
μ = 0.09 mm^−1^
*T* = 150 K
0.50 × 0.45 × 0.25 mm

#### Data collection

Stoe IPDS II diffractometer
38412 measured reflections
5433 independent reflections
4254 reflections with *I* > 2σ(*I*)
*R*_int_ = 0.045

#### Refinement

*R*[*F* ^2^ > 2σ(*F* ^2^)] = 0.029
*wR*(*F* ^2^) = 0.053
*S* = 0.86
5433 reflections
271 parameters
H-atom parameters constrained
Δρ_max_ = 0.13 e Å^−3^
Δρ_min_ = −0.18 e Å^−3^

### 

Data collection: *X-AREA* (Stoe & Cie, 2005 ▶); cell refinement: *X-AREA*; data reduction: *X-AREA*; program(s) used to solve structure: *SHELXS97* (Sheldrick, 2008 ▶); program(s) used to refine structure: *SHELXL97* (Sheldrick, 2008 ▶); molecular graphics: *XP* in *SHELXTL* (Sheldrick, 2008 ▶); software used to prepare material for publication: *SHELXL97*.

## Supplementary Material

Crystal structure: contains datablocks I, global. DOI: 10.1107/S1600536811014061/om2419sup1.cif

Structure factors: contains datablocks I. DOI: 10.1107/S1600536811014061/om2419Isup2.hkl

Additional supplementary materials:  crystallographic information; 3D view; checkCIF report

## Figures and Tables

**Fig. 1.**
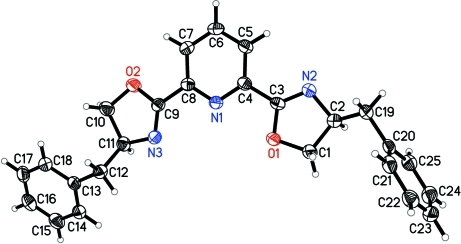
The molecular structure of the title compound showing the atom-labelling scheme. Displacement ellipsoids are drawn at the 50% probability level.

## supplementary crystallographic information

### Comment

The synthesis of chiral tridentate *N*,*N*,*N*-pyridine-2,6-bisoxazolines (pybox ligands) constitute a useful toolbox for the application in asymmetric catalysis (Desimoni *et al.*, 2003). The title compound was used as part of our ongoing studies (Tse *et al.*, 2006).

In contrast to the complexed ligand (Nesper *et al.*, 1996; Provent *et al.*, 1997) where all N atoms are pointed to the metal to permit coordination, in the free ligand the N atoms of both oxazoline rings point in the opposite direction (Fig. 1). The dihedral angle between the planes defined by C13 - C18 and C20 - C25 is 72.85 (3)°. The phenyl rings (C13 - C18 and C20 - C25) are twisted out of the N1, C4 - C8 plane by an angle of 30.56 (5)° and 84.57 (3)°, respectively. The absolute configuration has been assigned to correspond with that of the known chiral centres of the starting material.

### Experimental

The synthesis of the commercially available title compound was described by Nesper *et al.*, 1993; Nesper *et al.*, 1996; Schaus & Jacobsen, 2000; Towers *et al.*, 2003; Meng *et al.*, 2005 and Hui *et al.*, 2006. The title compound was purchased from STREM and crystals were grown from a dichloromethane/hexane mixture. The solution was slowly evaporated to dryness for two days and colourless crystals suitable for X-ray analysis were isolated.

### Refinement

H atoms were placed in idealized positions with d(C—H) = 0.99 (CH_2_) and 0.95–1.00 Å (CH) and refined using a riding model with *U*_iso_(H) fixed at 1.2 *U*_eq_(C).

### Crystal data

**Table:**

C_25_H_23_N_3_O_2_	*F*(000) = 840
*M**_r_* = 397.46	*D*_x_ = 1.316 Mg m^−^^3^
Orthorhombic, *P*2_1_2_1_2_1_	Mo *K*α radiation, λ = 0.71073 Å
Hall symbol: P 2ac 2ab	Cell parameters from 7372 reflections
*a* = 7.0184 (2) Å	θ = 1.8–29.6°
*b* = 13.2654 (3) Å	µ = 0.09 mm^−^^1^
*c* = 21.5542 (8) Å	*T* = 150 K
*V* = 2006.74 (10) Å^3^	Prism, colourless
*Z* = 4	0.50 × 0.45 × 0.25 mm

### Data collection

**Table:**

Stoe IPDS II diffractometer	4254 reflections with *I* > 2σ(*I*)
Radiation source: fine-focus sealed tube	*R*_int_ = 0.045
graphite	θ_max_ = 29.2°, θ_min_ = 1.8°
ω scans	*h* = −9→9
38412 measured reflections	*k* = −18→18
5433 independent reflections	*l* = −29→29

### Refinement

**Table:**

Refinement on *F*^2^	Primary atom site location: structure-invariant direct methods
Least-squares matrix: full	Secondary atom site location: difference Fourier map
*R*[*F*^2^ > 2σ(*F*^2^)] = 0.029	Hydrogen site location: inferred from neighbouring sites
*wR*(*F*^2^) = 0.053	H-atom parameters constrained
*S* = 0.86	*w* = 1/[σ^2^(*F*_o_^2^) + (0.0277*P*)^2^] where *P* = (*F*_o_^2^ + 2*F*_c_^2^)/3
5433 reflections	(Δ/σ)_max_ = 0.001
271 parameters	Δρ_max_ = 0.13 e Å^−^^3^
0 restraints	Δρ_min_ = −0.18 e Å^−^^3^

### Special details

**Table:**

Geometry. All e.s.d.'s (except the e.s.d. in the dihedral angle between two l.s. planes) are estimated using the full covariance matrix. The cell e.s.d.'s are taken into account individually in the estimation of e.s.d.'s in distances, angles and torsion angles; correlations between e.s.d.'s in cell parameters are only used when they are defined by crystal symmetry. An approximate (isotropic) treatment of cell e.s.d.'s is used for estimating e.s.d.'s involving l.s. planes.
Refinement. Refinement of *F*^2^ against ALL reflections. The weighted *R*-factor *wR* and goodness of fit *S* are based on *F*^2^, conventional *R*-factors *R* are based on *F*, with *F* set to zero for negative *F*^2^. The threshold expression of *F*^2^ > σ(*F*^2^) is used only for calculating *R*-factors(gt) *etc*. and is not relevant to the choice of reflections for refinement. *R*-factors based on *F*^2^ are statistically about twice as large as those based on *F*, and *R*- factors based on ALL data will be even larger.

### Fractional atomic coordinates and isotropic or equivalent isotropic displacement parameters (Å^2^)

**Table:**

	*x*	*y*	*z*	*U*_iso_*/*U*_eq_	
C1	0.08221 (18)	0.78242 (8)	0.86378 (6)	0.0391 (3)	
H1A	0.0477	0.8470	0.8436	0.047*	
H1B	0.1124	0.7953	0.9079	0.047*	
C2	−0.07905 (16)	0.70557 (7)	0.85760 (5)	0.0307 (2)	
H2	−0.1664	0.7274	0.8235	0.037*	
C3	0.18890 (16)	0.63775 (7)	0.82495 (5)	0.0271 (2)	
C4	0.33449 (15)	0.56927 (7)	0.79877 (5)	0.0271 (2)	
C5	0.30631 (16)	0.46567 (7)	0.80236 (5)	0.0320 (2)	
H5	0.1993	0.4390	0.8238	0.038*	
C6	0.43561 (16)	0.40269 (8)	0.77450 (5)	0.0330 (3)	
H6	0.4199	0.3316	0.7762	0.040*	
C7	0.58872 (16)	0.44469 (7)	0.74402 (5)	0.0302 (2)	
H7	0.6801	0.4029	0.7241	0.036*	
C8	0.60768 (16)	0.54896 (7)	0.74277 (5)	0.0261 (2)	
C9	0.76549 (16)	0.59535 (7)	0.70744 (4)	0.0274 (2)	
C10	1.02808 (19)	0.58556 (8)	0.64867 (6)	0.0410 (3)	
H10A	1.0471	0.5688	0.6044	0.049*	
H10B	1.1483	0.5731	0.6715	0.049*	
C11	0.96205 (16)	0.69563 (8)	0.65652 (5)	0.0307 (2)	
H11	0.9089	0.7199	0.6161	0.037*	
C12	1.11831 (16)	0.76752 (8)	0.67706 (5)	0.0308 (2)	
H12A	1.0595	0.8305	0.6927	0.037*	
H12B	1.1903	0.7366	0.7116	0.037*	
C13	1.25418 (16)	0.79284 (7)	0.62524 (5)	0.0287 (2)	
C14	1.23770 (17)	0.88259 (8)	0.59240 (5)	0.0341 (2)	
H14	1.1407	0.9293	0.6032	0.041*	
C15	1.36016 (18)	0.90471 (10)	0.54433 (6)	0.0422 (3)	
H15	1.3472	0.9665	0.5225	0.051*	
C16	1.50168 (19)	0.83760 (9)	0.52768 (6)	0.0415 (3)	
H16	1.5855	0.8528	0.4944	0.050*	
C17	1.52003 (17)	0.74822 (9)	0.55991 (6)	0.0403 (3)	
H17	1.6169	0.7016	0.5488	0.048*	
C18	1.39795 (17)	0.72647 (8)	0.60832 (5)	0.0352 (3)	
H18	1.4126	0.6650	0.6304	0.042*	
C19	−0.19380 (17)	0.69334 (8)	0.91699 (5)	0.0338 (2)	
H19A	−0.3029	0.6477	0.9093	0.041*	
H19B	−0.1127	0.6623	0.9494	0.041*	
C20	−0.26647 (15)	0.79374 (7)	0.93971 (5)	0.0284 (2)	
C21	−0.19421 (16)	0.83776 (8)	0.99302 (5)	0.0331 (3)	
H21	−0.0999	0.8031	1.0164	0.040*	
C22	−0.25669 (18)	0.93129 (8)	1.01295 (5)	0.0369 (3)	
H22	−0.2051	0.9603	1.0496	0.044*	
C23	−0.39392 (18)	0.98226 (8)	0.97955 (6)	0.0385 (3)	
H23	−0.4370	1.0466	0.9930	0.046*	
C24	−0.46803 (17)	0.93922 (9)	0.92658 (6)	0.0380 (3)	
H24	−0.5632	0.9739	0.9037	0.046*	
C25	−0.40519 (17)	0.84613 (8)	0.90655 (5)	0.0329 (2)	
H25	−0.4570	0.8175	0.8698	0.040*	
N1	0.48293 (13)	0.61177 (6)	0.76977 (4)	0.02706 (19)	
N2	0.02005 (14)	0.61231 (6)	0.83845 (4)	0.0311 (2)	
N3	0.80484 (13)	0.68788 (6)	0.70200 (4)	0.0307 (2)	
O1	0.24182 (12)	0.73528 (5)	0.83241 (3)	0.03401 (18)	
O2	0.87275 (12)	0.52786 (5)	0.67492 (4)	0.0402 (2)	

### Atomic displacement parameters (Å^2^)

**Table:**

	*U*^11^	*U*^22^	*U*^33^	*U*^12^	*U*^13^	*U*^23^
C1	0.0401 (7)	0.0291 (5)	0.0482 (7)	−0.0040 (5)	0.0204 (6)	−0.0039 (5)
C2	0.0318 (6)	0.0266 (5)	0.0337 (6)	−0.0005 (4)	0.0013 (5)	0.0007 (4)
C3	0.0318 (6)	0.0241 (5)	0.0255 (5)	−0.0043 (4)	0.0004 (5)	0.0029 (4)
C4	0.0289 (6)	0.0273 (5)	0.0251 (5)	−0.0027 (4)	−0.0007 (4)	0.0015 (4)
C5	0.0320 (6)	0.0283 (5)	0.0357 (6)	−0.0042 (5)	0.0018 (5)	0.0048 (4)
C6	0.0398 (7)	0.0218 (5)	0.0375 (6)	−0.0016 (5)	−0.0023 (5)	0.0023 (4)
C7	0.0332 (6)	0.0264 (5)	0.0310 (6)	0.0034 (4)	0.0003 (5)	−0.0002 (4)
C8	0.0289 (6)	0.0249 (5)	0.0245 (5)	−0.0005 (4)	−0.0013 (4)	0.0006 (4)
C9	0.0293 (6)	0.0266 (5)	0.0262 (5)	0.0016 (5)	0.0004 (5)	−0.0035 (4)
C10	0.0403 (7)	0.0347 (6)	0.0480 (7)	−0.0067 (5)	0.0162 (6)	−0.0078 (5)
C11	0.0302 (6)	0.0337 (5)	0.0282 (5)	−0.0001 (5)	0.0037 (5)	0.0020 (4)
C12	0.0330 (6)	0.0295 (5)	0.0299 (5)	0.0017 (5)	0.0008 (5)	0.0009 (4)
C13	0.0255 (6)	0.0297 (5)	0.0310 (5)	−0.0031 (4)	−0.0036 (5)	−0.0033 (4)
C14	0.0294 (6)	0.0325 (5)	0.0404 (6)	−0.0010 (5)	−0.0008 (5)	0.0025 (4)
C15	0.0402 (7)	0.0439 (7)	0.0424 (7)	−0.0079 (6)	−0.0013 (6)	0.0104 (5)
C16	0.0349 (7)	0.0544 (7)	0.0351 (6)	−0.0136 (6)	0.0054 (6)	−0.0025 (5)
C17	0.0264 (6)	0.0450 (7)	0.0494 (7)	−0.0017 (5)	0.0039 (6)	−0.0128 (5)
C18	0.0297 (6)	0.0315 (6)	0.0444 (7)	−0.0004 (5)	−0.0007 (5)	−0.0013 (5)
C19	0.0294 (6)	0.0292 (5)	0.0429 (6)	−0.0006 (5)	0.0083 (5)	0.0054 (5)
C20	0.0228 (5)	0.0304 (5)	0.0320 (5)	−0.0024 (5)	0.0068 (5)	0.0071 (4)
C21	0.0263 (6)	0.0406 (6)	0.0323 (6)	0.0000 (5)	0.0018 (5)	0.0084 (4)
C22	0.0376 (7)	0.0416 (6)	0.0314 (5)	−0.0076 (5)	0.0043 (5)	−0.0013 (5)
C23	0.0416 (7)	0.0297 (6)	0.0443 (7)	0.0022 (5)	0.0132 (6)	0.0028 (5)
C24	0.0311 (6)	0.0396 (6)	0.0433 (7)	0.0069 (5)	0.0001 (5)	0.0101 (5)
C25	0.0289 (6)	0.0364 (6)	0.0335 (6)	−0.0028 (5)	−0.0015 (5)	0.0031 (5)
N1	0.0295 (5)	0.0247 (4)	0.0270 (4)	−0.0009 (4)	0.0019 (4)	−0.0001 (3)
N2	0.0297 (5)	0.0268 (4)	0.0370 (5)	−0.0031 (4)	0.0034 (4)	−0.0014 (4)
N3	0.0300 (5)	0.0277 (4)	0.0345 (5)	0.0021 (4)	0.0052 (4)	0.0030 (4)
O1	0.0351 (4)	0.0253 (3)	0.0416 (4)	−0.0049 (3)	0.0136 (4)	−0.0037 (3)
O2	0.0407 (5)	0.0283 (4)	0.0515 (5)	−0.0052 (4)	0.0187 (4)	−0.0111 (3)

### Geometric parameters (Å, °)

**Table:**

C1—O1	1.4503 (14)	C12—C13	1.5065 (15)
C1—C2	1.5290 (17)	C12—H12A	0.9900
C1—H1A	0.9900	C12—H12B	0.9900
C1—H1B	0.9900	C13—C18	1.3879 (15)
C2—N2	1.4781 (14)	C13—C14	1.3899 (14)
C2—C19	1.5211 (15)	C14—C15	1.3778 (16)
C2—H2	1.0000	C14—H14	0.9500
C3—N2	1.2660 (14)	C15—C16	1.3813 (18)
C3—O1	1.3556 (11)	C15—H15	0.9500
C3—C4	1.4790 (14)	C16—C17	1.3801 (17)
C4—N1	1.3394 (13)	C16—H16	0.9500
C4—C5	1.3905 (14)	C17—C18	1.3808 (16)
C5—C6	1.3719 (15)	C17—H17	0.9500
C5—H5	0.9500	C18—H18	0.9500
C6—C7	1.3773 (15)	C19—C20	1.5078 (14)
C6—H6	0.9500	C19—H19A	0.9900
C7—C8	1.3898 (13)	C19—H19B	0.9900
C7—H7	0.9500	C20—C21	1.3852 (15)
C8—N1	1.3415 (13)	C20—C25	1.3934 (15)
C8—C9	1.4784 (15)	C21—C22	1.3843 (16)
C9—N3	1.2636 (13)	C21—H21	0.9500
C9—O2	1.3636 (12)	C22—C23	1.3796 (17)
C10—O2	1.4472 (14)	C22—H22	0.9500
C10—C11	1.5413 (15)	C23—C24	1.3784 (17)
C10—H10A	0.9900	C23—H23	0.9500
C10—H10B	0.9900	C24—C25	1.3806 (15)
C11—N3	1.4796 (14)	C24—H24	0.9500
C11—C12	1.5193 (16)	C25—H25	0.9500
C11—H11	1.0000		
			
O1—C1—C2	104.10 (8)	C11—C12—H12B	109.1
O1—C1—H1A	110.9	H12A—C12—H12B	107.9
C2—C1—H1A	110.9	C18—C13—C14	118.04 (10)
O1—C1—H1B	110.9	C18—C13—C12	120.90 (9)
C2—C1—H1B	110.9	C14—C13—C12	121.05 (10)
H1A—C1—H1B	109.0	C15—C14—C13	120.90 (11)
N2—C2—C19	113.26 (8)	C15—C14—H14	119.6
N2—C2—C1	103.54 (9)	C13—C14—H14	119.6
C19—C2—C1	112.94 (9)	C14—C15—C16	120.44 (11)
N2—C2—H2	109.0	C14—C15—H15	119.8
C19—C2—H2	109.0	C16—C15—H15	119.8
C1—C2—H2	109.0	C17—C16—C15	119.34 (11)
N2—C3—O1	118.92 (10)	C17—C16—H16	120.3
N2—C3—C4	124.80 (9)	C15—C16—H16	120.3
O1—C3—C4	116.24 (9)	C16—C17—C18	120.13 (11)
N1—C4—C5	123.55 (10)	C16—C17—H17	119.9
N1—C4—C3	117.18 (8)	C18—C17—H17	119.9
C5—C4—C3	119.17 (10)	C17—C18—C13	121.14 (10)
C6—C5—C4	118.91 (10)	C17—C18—H18	119.4
C6—C5—H5	120.5	C13—C18—H18	119.4
C4—C5—H5	120.5	C20—C19—C2	110.99 (8)
C5—C6—C7	118.60 (9)	C20—C19—H19A	109.4
C5—C6—H6	120.7	C2—C19—H19A	109.4
C7—C6—H6	120.7	C20—C19—H19B	109.4
C6—C7—C8	119.11 (10)	C2—C19—H19B	109.4
C6—C7—H7	120.4	H19A—C19—H19B	108.0
C8—C7—H7	120.4	C21—C20—C25	118.10 (10)
N1—C8—C7	123.18 (10)	C21—C20—C19	121.19 (10)
N1—C8—C9	116.99 (8)	C25—C20—C19	120.69 (10)
C7—C8—C9	119.74 (9)	C22—C21—C20	121.29 (11)
N3—C9—O2	118.00 (9)	C22—C21—H21	119.4
N3—C9—C8	128.02 (9)	C20—C21—H21	119.4
O2—C9—C8	113.89 (8)	C23—C22—C21	119.90 (11)
O2—C10—C11	103.39 (9)	C23—C22—H22	120.1
O2—C10—H10A	111.1	C21—C22—H22	120.1
C11—C10—H10A	111.1	C24—C23—C22	119.52 (11)
O2—C10—H10B	111.1	C24—C23—H23	120.2
C11—C10—H10B	111.1	C22—C23—H23	120.2
H10A—C10—H10B	109.0	C23—C24—C25	120.59 (11)
N3—C11—C12	112.88 (9)	C23—C24—H24	119.7
N3—C11—C10	103.36 (8)	C25—C24—H24	119.7
C12—C11—C10	114.18 (10)	C24—C25—C20	120.60 (11)
N3—C11—H11	108.7	C24—C25—H25	119.7
C12—C11—H11	108.7	C20—C25—H25	119.7
C10—C11—H11	108.7	C4—N1—C8	116.66 (8)
C13—C12—C11	112.38 (9)	C3—N2—C2	106.35 (8)
C13—C12—H12A	109.1	C9—N3—C11	106.97 (8)
C11—C12—H12A	109.1	C3—O1—C1	104.79 (8)
C13—C12—H12B	109.1	C9—O2—C10	105.64 (8)
